# Electrical Switching in Semiconductor-Metal Self-Assembled VO_2_ Disordered Metamaterial Coatings

**DOI:** 10.1038/srep37699

**Published:** 2016-11-24

**Authors:** Sunil Kumar, Francis Maury, Naoufal Bahlawane

**Affiliations:** 1 Luxembourg Institute of Science and Technology (LIST), 5 avenue des Hauts-Fourneaux L-4362 Esch-sur-Alzette Luxembourg; 2 CIRIMAT, ENSIACET-4 allée E. Monso, 31030 Toulouse, France

## Abstract

As a strongly correlated metal oxide, VO_2_ inspires several highly technological applications. The challenging reliable wafer-scale synthesis of high quality polycrystalline VO_2_ coatings is demonstrated on 4” Si taking advantage of the oxidative sintering of chemically vapor deposited VO_2_ films. This approach results in films with a semiconductor-metal transition (SMT) quality approaching that of the epitaxial counterpart. SMT occurs with an abrupt electrical resistivity change exceeding three orders of magnitude with a narrow hysteresis width. Spatially resolved infrared and Raman analyses evidence the self-assembly of VO_2_ disordered metamaterial, compresing monoclinic (M1 and M2) and rutile (R) domains, at the transition temperature region. The M2 mediation of the M1-R transition is spatially confined and related to the localized strain-stabilization of the M2 phase. The presence of the M2 phase is supposed to play a role as a minor semiconducting phase far above the SMT temperature. In terms of application, we show that the VO_2_ disordered self-assembly of M and R phases is highly stable and can be thermally triggered with high precision using short heating or cooling pulses with adjusted strengths. Such a control enables an accurate and tunable thermal control of the electrical switching.

The property of exhibiting phase transitions in strongly correlated metal oxides has opened up new application possibilities. Vanadium dioxide (VO_2_) has seen a particular interest owing to its Semiconductor to Metal Transition (SMT) occurring at a temperature (T_c_) of 67 °C^1^. VO_2_ undergoes a first order phase transition from a highly resistive semiconducting monoclinic (M1) phase to a metallic rutile (R) phase with an effective change of electrical resistivity of 3–4 orders of magnitude within a narrow range of temperature^2^,^3^,^4^. This change in electrical properties is accompanied by a remarkable optical transition, where the material shows thermochromic behavior in the infrared (IR) region. VO_2_ effectively reflects the IR radiation in the high temperature rutile phase while being IR transparent at the low temperature monoclinic phase^5^,^6^,^7^,^8^. Such unique combination of properties marks it as a crucial material of study not only for developing intelligent thermal, resistive and optical switches^9^,^10^,^11^,^12^,^13^,^14^,^15^, but also from the fundamental point of view.

Obtaining high quality VO_2_ films, essential for high technological applications, remains a challenge as vanadium forms multiple stable oxides like V_2_O_3_, V_2_O_5_ and V_6_O_13_^16^. Hence, it is crucial to control the growth conditions to a high degree of precision to obtain pure single-phase films. Polycrystalline and epitaxial films have been grown by various deposition techniques including Sol gel^17^, Pulsed Laser Deposition (PLD)^4^, Molecular Beam Epitaxy (MBE)^18^, Atomic Layer Deposition (ALD)^19^, Sputtering^20^, and Chemical Vapor Deposition (CVD)^21^,^22^,^23^. Epitaxial films grow on pre-treated and appropriately oriented Al_2_O_3_ or TiO_2_ substrates or buffer layers^24^,^25^,^26^. The SMT-relevant indicators of the VO_2_ films quality are the amplitude of resistivity change and hysteresis width. Ideally, VO_2_ films show 3–4 orders of magnitude resistivity change with a narrow hysteresis width of ΔT~ 3–4 K. Bulk single crystal offers slightly higher amplitude of resistivity change but is susceptible to breakdown after few cycles of switching between semiconducting and metallic phases^27^. Although, polycrystalline thin films withstand frequent cycling, they usually feature broad hysteresis and small amplitudes of resistivity change, due to the presence of high density of grain boundaries and grain-boundary defects^23^,^28^,^29^. Sintering as-grown VO_2_ films to decrease the density of grain boundaries is not conceivable for practical applications due to the high melting point of this phase (1970 °C). The depression of the melting point, usually observed in nano-crystalline materials^30^ is unlikely to reduce the sintering temperature to a reasonable range. Epitaxial VO_2_ films attracted a considerable attention owing to their improved morphological advantages and SMT quality. Nevertheless, cost, process conditions, ease of synthesis, morphological control and industrial integration remain limiting challenges for epitaxial growth of VO_2_. Hence, it is worth investigating ways to grow polycrystalline VO_2_ films without specific buffer layers, yet still matching the performance of epitaxial or single crystal VO_2_.

The synthesis of high quality, electronic grade, wafer-scale VO_2_ films by MOCVD is achieved involving an oxidative sintering step. The investigation of the electrical and optical properties across the SMT reveals the self-assembly of a VO_2_–disordered metamaterial in which the coalescence and confinement of metallic domains are highly controllable.

## Results and Discussion

### As-deposited films

**Figure 1:**
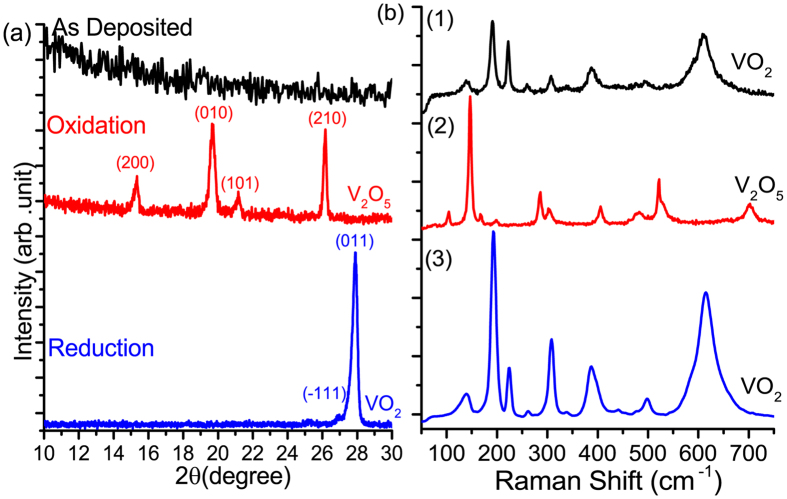
XRD patterns (**a**) and Raman spectra (**b**) of (1) as-grown film, (2) pure phase orthorhombic V_2_O_5_ (PDF no-750457) obtained after oxidation and (3) monoclinic VO_2_ M1 phase (PDF no-03-065-2358) obtained upon V_2_O_5_ annealing under vacuum. The average crystallite size of V_2_O_5_ and VO_2_ is 22 nm and 27 nm respectively.

The as-grown films using cyclohexane as a liquid carrier (step 1) are XRD-amorphous (Fig. 1a), which contrasts with the crystalline VO_2_ films obtained with ethanol at this temperature range^23^. On the other hand, cyclohexane is thermally more stable than ethanol at 600 °C^31^. For instance, the pyrolysis of ethanol in the temperature range 576–624 °C produces essentially methane, hydrogen and oxygen containing compounds as acetaldehyde and carbon monoxide^32^. In contrast to cyclohexane, ethanol is able to participate into the deposition chemistry as a potential source of oxygen. It is worth mentioning that the as-grown films represent the VO_2_ characteristic Raman signature (Fig. 1b) but do not feature any obvious sudden change of electrical resistivity upon heating. It is therefore necessary to apply post-deposition treatments to improve the crystallinity of VO_2_ films and decrease the density of the grain boundaries.

#### Post deposition thermal treatment

**Figure 2:**
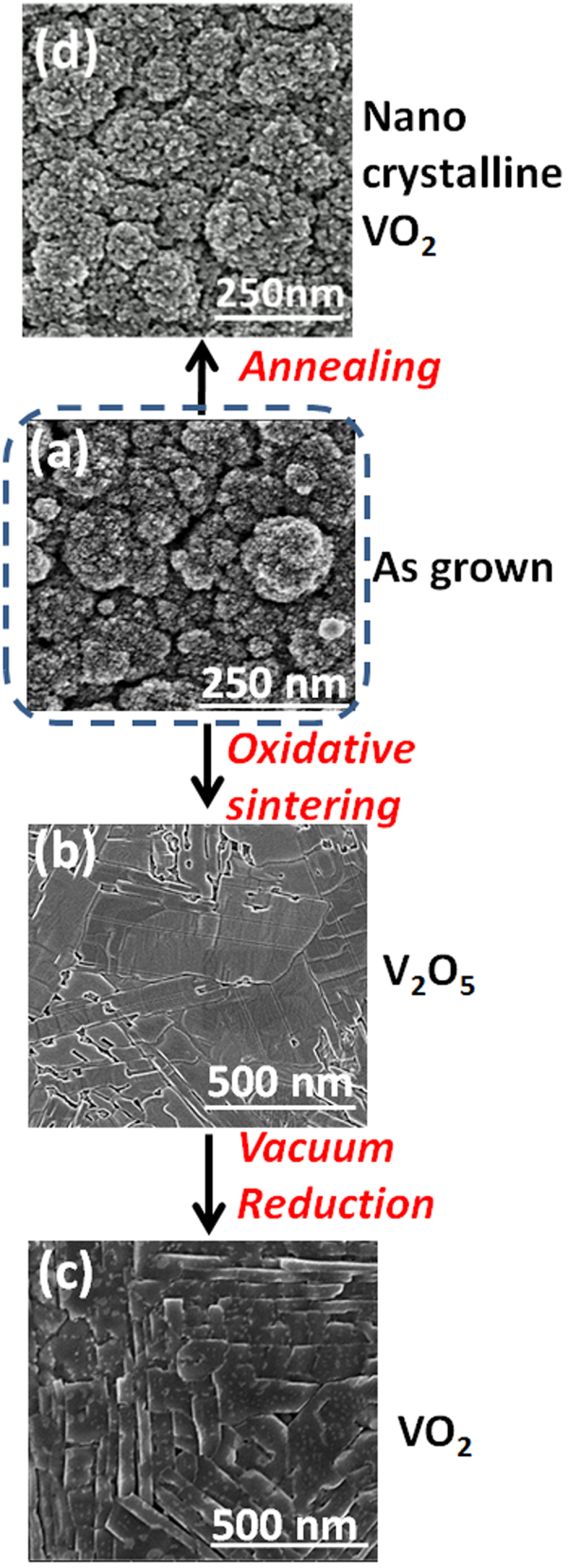
Scanning electron micrographs showing the evolution of film morphology at different stages of film processing from (**a**) the as-grown amorphous vanadium oxide film, (**b**) sintered V_2_O_5_ film to a (**c**) sintered VO_2_ by vacuum reduction. 4 hours annealing of VO_2_ at 600 °C under vacuum induces a marginal morphological impact (**d**). The film thickness is 500 nm.

Two approaches were implemented to induce the sintering of VO_2_ films. Annealing under vacuum in the absence of oxygen was performed at 600 °C directly in the deposition chamber. As this temperature is far below the melting point (1970 °C) of VO_2_, no significant sintering took place as shown in Fig. 2(a–d). The second approach involves the conversion of VO_2_ to V_2_O_5_, that exhibits a lower melting point (690 °C), prior sintering. This approach proves to be successful as displayed in Fig. 2(a,b). The conversion of VO_2_ to V_2_O_5_ was performed under the O_2_ partial pressure of 0.01 mbar. The XRD analysis, supplementary information (S1), shows the occurrence of the VO_2_ – V_2_O_5_ conversion already at 400 °C. Fixing the temperature at 600 °C was essentially implemented to induce an efficient sintering over a short period (1 h) and to simplify this multi-step process by keeping the substrate temperature constant.

The sintered V_2_O_5_ films undergo reduction to VO_2_ at the same temperature in the absence of oxygen as shown in Figs 1 and 2. The surface micrographs, Fig. 2a–c, display the evolution of the film microstructure at the various steps of the process from porous nano grains to large and well-shaped domains. Upon reduction under vacuum, V_2_O_5_ releases oxygen without significantly affecting the obtained dense morphology (Fig. 2b,c). An extended treatment under these conditions is expected to yield V_2_O_3_^33^.

XRD and Raman scattering, Fig. 1, indicate the nano-crystalline nature of the as-grown VO_2_ films that are XRD-amorphous but feature the characteristic VO_2_ Raman peaks. The oxidative sintering step yields an orthorhombic V_2_O_5_ that converts into crystalline VO_2_ (M_1_) after further annealing under vacuum. Raman spectrum of the crystalline VO_2_ features an enhanced scattering intensity.

#### Film properties

**Figure 3:**
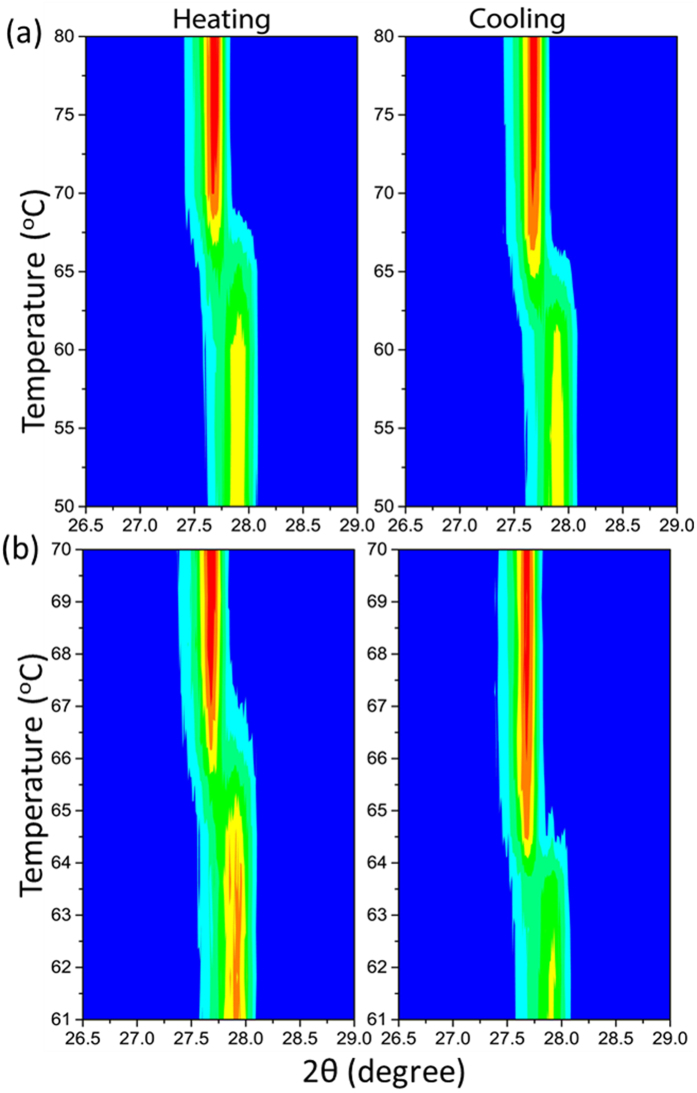
Contour plots of X-ray diffraction during the structural phase transition occurring during heating and cooling stages in the ranges: 50 to 80 °C (**a**) and 61–70 °C (**b**).

Temperature-programmed X- ray diffraction was carried from room temperature up to 130 °C. Contour plots shown in Fig. 3 point towards an abrupt change of the diffractogram during heating and cooling cycles. Figure 3(a) shows the phase transition of VO_2_ in the 50–80 °C range whereas Fig. 3(b) presents a closer look at the changes taking place near the structural phase transition in the 61–70 °C range. The peak at 2θ = 27.9° corresponding to (011), vanishes abruptly at 65–66 °C in the heating cycle. Above this temperature a diffraction peak at 2θ = 27.6° is suddenly detected. The reverse transition occurs at 63–64 °C upon cooling, which reveals a narrow hysteresis width of ~1–2 K. Further detailed X-ray diffraction data can be found in the supplementary section (Figure S2).

**Figure 4:**
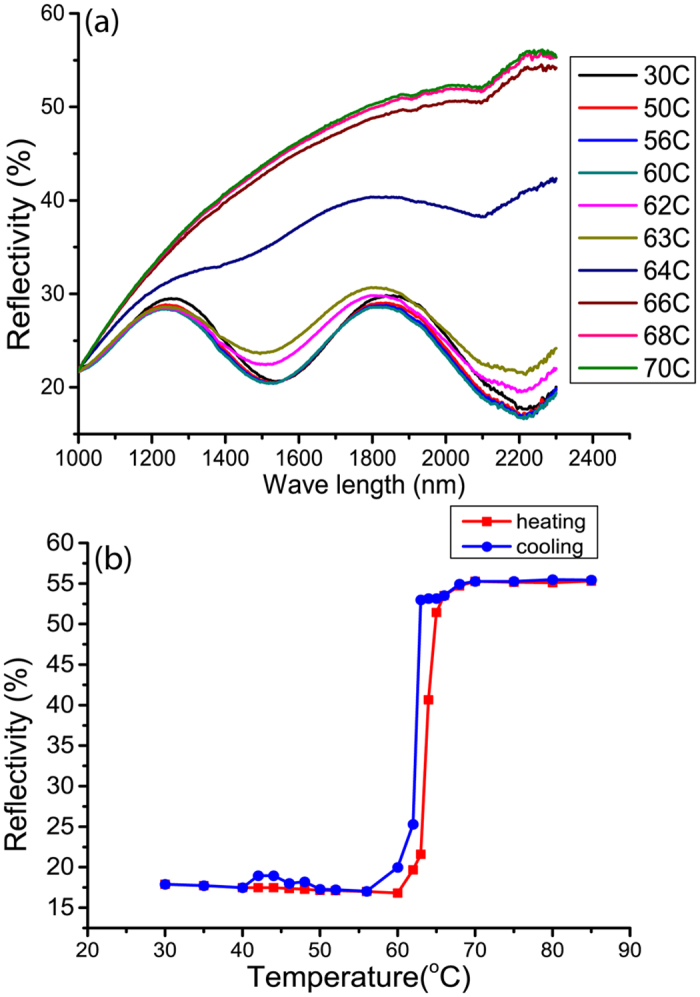
Temperature-dependent infrared reflectivity in the NIR region: Reflection spectra in the NIR upon heating (**a**) and the variation in reflection across the transition temperature (**b**) displayed for the arbitrary selected wavelength λ = 2300 nm.

The optical reflectivity and thermal imaging were acquired as a function of temperature for 500 nm-thick VO_2_ films. The wavelength-dependent total hemispherical reflections in the near infrared region (NIR) are displayed in Fig. 4 across the SMT. The thermochromic behavior is clearly shown by an abrupt increase of the reflection from e.g. 18% to 55% at λ = 2300 nm by increasing the temperature. Figure 4(b) shows the reflection hysteresis curve at this wavelength. The observed sharp transition and narrow hysteresis, which agree with the XRD results, are relevant assets for energy efficient glazing and static solar control applications^10^,^12^,^34^,^35^.

Based on the spectroscopic measurements it is clear that the monoclinic and rutile phases of VO_2_ feature a contrasting thermal emissivity. This contrast was represented in the NIR imaging to spatially resolve the phase transition across the SMT. The surface temperature is captured using a neighboring coated silicon with a thick (4–5 μm) carbon nanotube layer which acts as a perfect black body.

**Figure 5:**
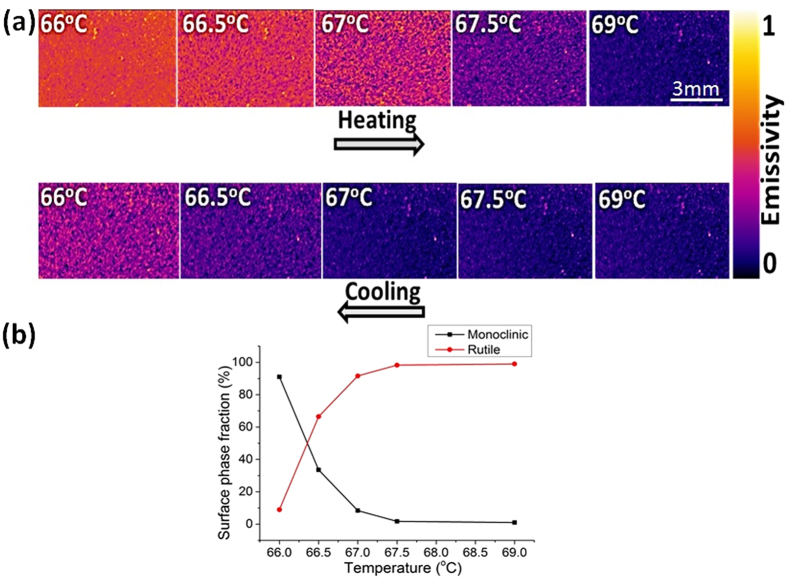
(**a**) Thermal imaging of VO_2_ film near the phase transition showing the formation of small metallic clusters as purple spots that grow in size with temperature. (**b**) The fraction of monoclinic and rutile phases calculated from the change of color in the thermal images during the cooling stage.

NIR images in Fig. 5a display the evolution of the rutile metallic domains (low emissivity) with increasing temperature across the transition. These domains grow rapidly in size with a small increase in temperature until coalescence. Similar to temperature dependent XRD patterns and NIR reflection behavior, we notice a difference of ΔT = 1–2 K between the heating and cooling stages, which confirms the small width of the hysteresis curve. The fraction and distribution of the metallic domains at 67–67.5 °C during the heating stage are equivalent to these at 66–66.5 °C in the cooling stage.

In a finite range of temperature (66–69 °C), both semiconducting and metallic phases with contrasting electrical and optical properties co-exist. One of the early descriptions of VO_2_ in this range was proposed by H. S. Choi *et al*.^36^ which implemented the corresponding model to simulate the electrical conduction behavior. Later the same region of phase co-existence was also referred to as “monoclinic and correlated metal” (MCM)^37^ and “strongly correlated metal”^38^. The most recent description of this transition state of VO_2_ is “disordered metamaterial”^39^,^40^.

Yun Zhang *et al*.^41^ report on disordered metal nanoparticles (MNP) in a dielectric matrix, yet still featuring near perfect metamaterial absorption behavior. Authors successfully show that the order is not necessary to attain controllable metamaterials. Thus VO_2_ in a finite range of temperature features essentially the same kind of physical features as the aforementioned MNP in a dielectric matrix, which strengthens the recent identification of vanadium oxide transition state as “disordered metamaterial”.

The dominance of the metallic phase, Fig. 5(a), at 66.5 °C contrasts greatly between the heating and cooling stages. Upon heating, the film is overwhelmingly composed of monoclinic semiconducting phase, whereas, it shows mainly a rutile metallic phase upon cooling. Hence, the self-assembled disordered metamaterial in this temperature range features an appealing modular optical and electrical properties with high thermal sensitivity. The persisting small bright spots in the IR images correspond to surface defects as heterogeneities in surface topography (roughness, micron and submicron thickness, to name some) that influences the IR emissivity^42^.

The fraction of each phase changes abruptly, Fig. 5b, and the rutile domains grow in size and coalesce to form the majority phase at the transition temperature. The semiconducting and metallic domains co-exist and a small thermal excitation can result in large changes in terms of the self-assembly and the dominance of one phase over the other. The major change occurs essentially in a temperature window of ΔT = 1 K. On reaching up to 69 °C the film is completely dominated by the R phase, but there is still a tiny fraction of M phase.

**Figure 6:**
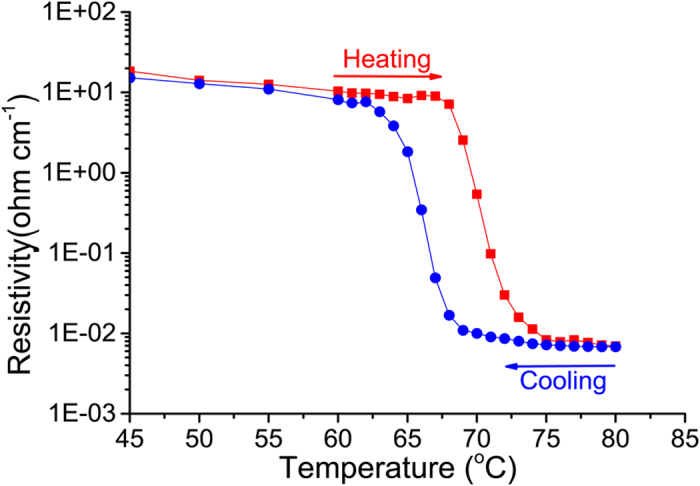
Change in electrical resistivity with temperature in heating and cooling cycles.

**Figure 7:**
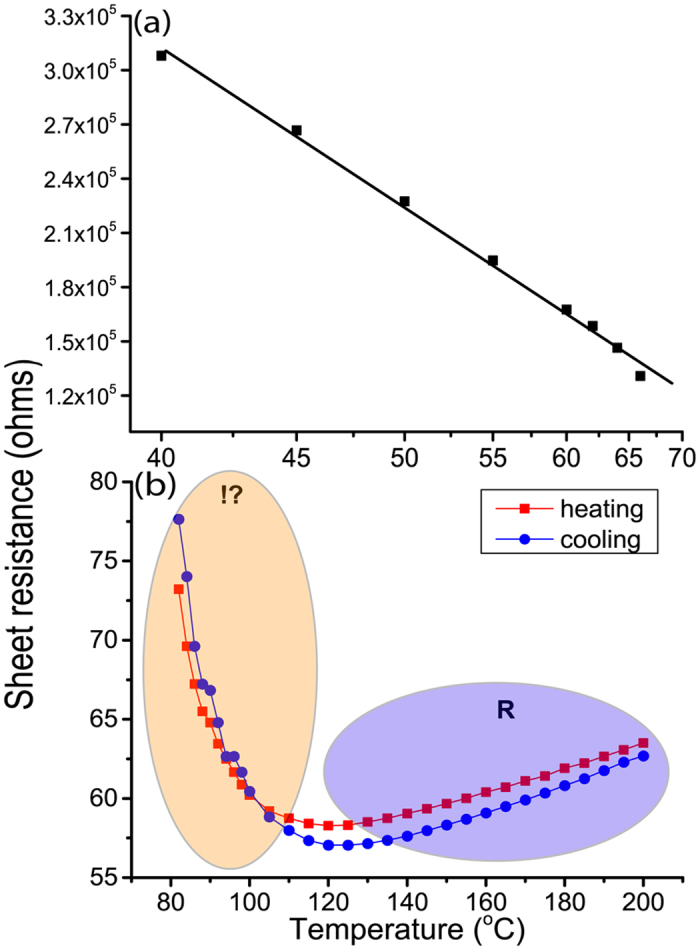
Electrical resistance in semiconducting monoclinic (M) and metallic rutile (R) phases of the VO_2_ film in the temperature ranges: (**a**) 40–65 °C and (**b**) 80–200 °C. Notice the further decrease in resistance long after the SMT with temperature in the cooling and heating stages.

Electrical resistivity was measured as a function of temperature, Fig. 6, for a 500 nm thick film on a Si substrate with its native oxide. The SMT induces an electrical resistivity change exceeding 3 orders of magnitude in the temperature range from 60 to 75 °C. The ratio of sheet resistances R(30 °C)/R(100 °C) gives a value of 0.66 10^4^ (see Fig. 7). The SMT occurs at 67 °C in the heating cycle and the reverse phase transition occurs at 64 °C indicating a hysteresis width of 3 K.

Electrical resistance of the film is a macroscopic property that essentially takes into account the percolation between the metallic domains or the kinetics of their formation, growth and coalescence. The collective behavior of the film is therefore not representative of the intrinsic microscopic SMT. In the microscopic regime, isolated metallic domains appear almost instantaneously throughout the film at a much lower temperature (a video is provided as supplementary materials: Figure S3). Besides the formation of new rutile nuclei upon the increase of temperature, the existing domains grow and coalesce^43^, forming electrically conductive paths by percolation. It is worth to note that the resistivity of VO_2_ depends on the coalescence of the metallic domains rather than their apparition. Therefore, the observed hysteresis for the electrical resistivity should be related to the temperature-dependent kinetics of coalescence or confinement of the metallic domains. The here observed hysteresis width, ΔT = 3 K, contrasts with the obtained width, ΔT = 1–2 K, from XRD (Fig. 3), IR reflection (Fig. 4) and thermal imaging (Fig. 5) measurements. The hysteresis in the last cases reflects rather the phase predominance and not the coalescence of the metallic rutile domains.

Further expanding on the electrical properties of the film, we can distinguish the electrical behavior of VO_2_ into two states, namely a high resistance semiconducting and low resistance metallic region at low and high temperatures respectively. Figure 7a exhibits the expected semiconducting behavior observed below 65 °C where the electrical resistivity decreases with temperature due to the thermal activation of charge carriers. The deduced temperature coefficient of resistance (TCR) and activation energy in the semiconducting region, −2.6% K^−1^ and 0.3 eV respectively, agree with values reported for VO_x_ films^44^,^45^,^46^. A linear increase of the electrical resistance, Fig. 7b, is observed with temperature above 120 °C indicating the metallic nature with a TCR of +0.17% K^−1^ that concurs with the reported values of common metals and alloys^47^. Interestingly films feature a metal behavior with positive TCR only starting at 120 °C, which is far above the SMT temperature.

The electrical resistance continues to decrease above the SMT with temperature, as displayed in Fig. 7b. This hints at the persistence of a semiconducting-like behavior due to competing contribution of the residual semiconducting phase resulting in a negative coefficient of resistance above the SMT. The occurrence of this behavior equally during the heating and the cooling stages, Fig. 7b, indicates its intrinsic nature. This has been noticed by Zhang *et al*.^48^ upon the investigation of the electrical properties of single VO_2_ nanobeam within the 70–110 °C temperature range. Authors could evidence the coexistence of M2 and R phases in this temperature range, and show their comparable electrical resistivity^49^. Although it seems to be overlooked, the negative TCR above SMT is visible in results displayed in several reports^29^,^44^,^49^. Jones *et al*.^50^ have reported the potential presence of M2 phase along with R at temperatures significantly higher than the SMT.

Regarding the M2 phase of VO_2_, Ji *et al*.^51^ have reported its presence in epitaxial VO_2_ and noticed a remarkable impact of the film’s strain on its dominance and stabilization temperature. Kim *et al*.^37^ have interestingly concluded that VO_2_ undergoes a two-step (M1-M2-R) conversion during the electrically driven transition, which is in line with static lattice calculations that predict an intermediate phase with a Peierls distortion when approaching the phase transition between M1 and the rutile phases^52^,^53^. A strong surface-induced stress stabilizes the M2 phase, which is assumed to mediate the transition^50^,^54^.

The presence of M2 would indeed clarify the observed semiconductor behavior after the first order phase transition at 70 °C. Thermal imaging, Fig. 5, does not allow distinguishing M1 from M2 phase because of their presumably similar emissivity values. Raman scattering, however, enables distinguishing these two phases via a significant A_g_ band shift from 620 cm^−1^ in M1 to 650 cm^−1^ in M2^51^. The Raman spectrum of the rutile metallic phase is devoid of any peaks. Temperature-programmed Raman spectroscopy was implemented to investigate the eventual formation of the M2 phase through the SMT. As displayed in the supplementary information document, Figure S4, the M2 intermediate phase could be detected, but not systematically even at micrometer distances on the same sample. This seemingly unreliable detection of the M2 phase hints at its localized formation. This observation would agree with the assumption of Ji *et al*.^51^ that the formation of M2 phase is likely related to the presence of tensile stress. It is noteworthy that reports addressing the detection of M2 phase implement high-resolution X-ray diffraction, tip enhanced Raman spectroscopy and localized strain modulation of single crystal nanobeams^37^,^54^,^55^.

Unlike nanobeams, such a stress might be randomly localized in polycrystalline films. Not detecting the M2 phase with XRD (Fig. 3), where the signal is averaged over an area of several square millimeters, indicates that M2 is a minor phase in the entire investigated temperature range.

**Figure 8:**
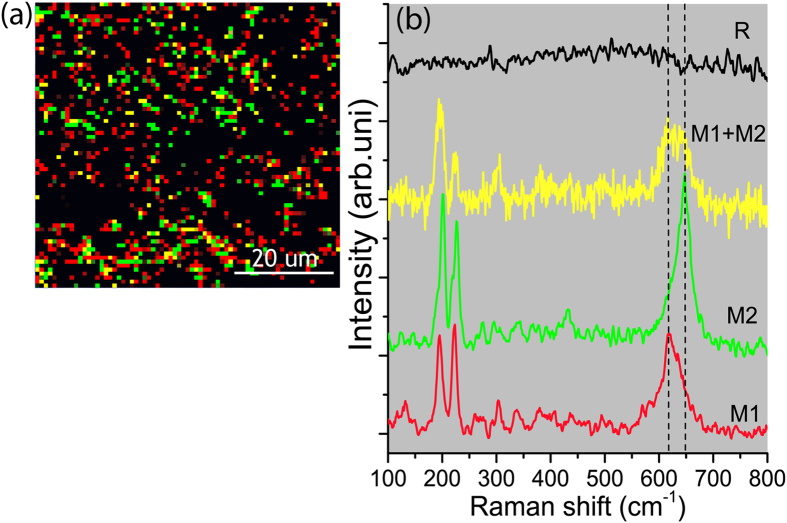
(**a**) Raman surface mapping of the M1, M2, M1 + M2 and R phases as measured at 67.5 °C (each dot correspond to 1 × 1 μm^2^ analysis area) and (**b**) Raman spectra corresponding to the color-coded used in the mapping.

Therefore, Raman mapping was performed at 67.5 °C for both wavenumbers (M2:650 cm^−1^ and M1:620 cm^−1^), Fig. 8a, to spatially localize the distribution of M1, M2 and R phases. This enables the spatial mapping of polycrystalline VO_2_ surface to visualize the random distribution of different phases. Each pixel corresponds to a 1 × 1 μm^2^ analysis area. The red dots in Fig. 8a represent M1 phase, whereas the green and yellow dots represent M2 and M1 + M2 domains respectively. The black background illustrates the presence of the R phase of VO_2_. The spectra of the aforementioned colored points in the Raman map are displayed in Fig. 8b. Hence, Raman mapping evidences clearly the self-assembly of a disordered M1-M2-R metamaterial at the transition temperature. It is worth mentioning that the presence of M2 phase was not evidenced above 70 °C, which is likely due to the excessive dominance of the metallic rutile phase. The entire SEM micrograph, Fig. 2, depicts a certain number of crystals and grain boundaries in an area that nearly corresponds to 1 × 1 μm^2^, which is the size of a single pixel in Fig. 8. This means that every single pixel (Fig. 8) contains a large number of grains/crystals and grain boundaries. Therefore, correlating the detection of M2 phase with morphological features such as grain boundary seems unlikely.

Based on the Raman mapping recorded at 67.5 °C, Fig. 8a, and the temperature-programmed Raman measurement, Figure S4 in the supplementary information, it could be shown that thermally driven phase transition in polycrystalline VO_2_ film involves indeed the formation of the M2 phase but in confined locations. It is assumed that localized strain in these confined areas meets conditions where M2 significantly forms as intermediate phase.

It is noteworthy that the film was maintained at this state at 67.5 °C in air for extended periods (100 hrs) without any apparent degradation or drift of one state to another. Therefore, the disordered VO_2_-metamaterial phase is quite stable and robust, which is an asset to its potential implementation in practical applications.

#### Vanadium oxide film as a thermally controlled electrical switch

As noticed during the investigation of the spatial phase distribution within VO_2_, the metamaterial phase is surprisingly stable over extended periods. Therefore, it is virtually possible to stabilize the system at any point within the hysteresis curve by adjusting the temperature. A practical application of this feature could be a thermally activated electrical switch, which might operate by the supply of short heating and cooling pulses with small amplitudes ΔT. These pulses bring the metamaterial to cycle between resistive “Off” and conductive “On” states while keeping the same background temperature around the SMT value.

**Figure 9:**
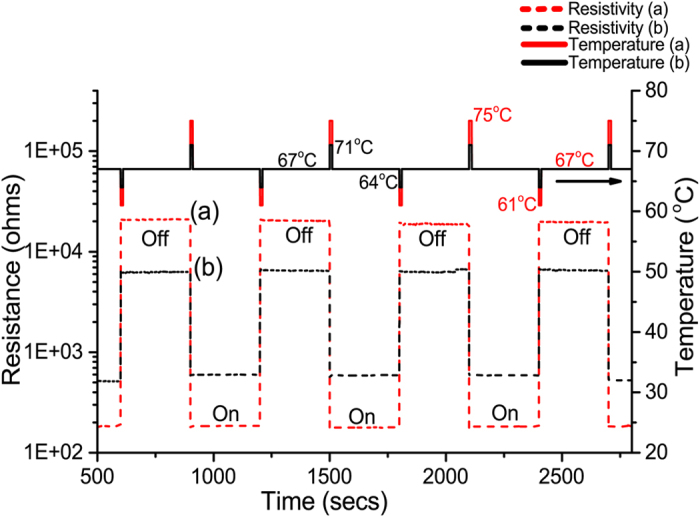
Thermal switching behavior of the VO_2_ disordered metamaterial. The “off” and “on” states are determined by the sudden drop or increase in electrical resistance as a consequence of small changes in the temperature given in the form of thermal activation pulses.

**Figure 10:**
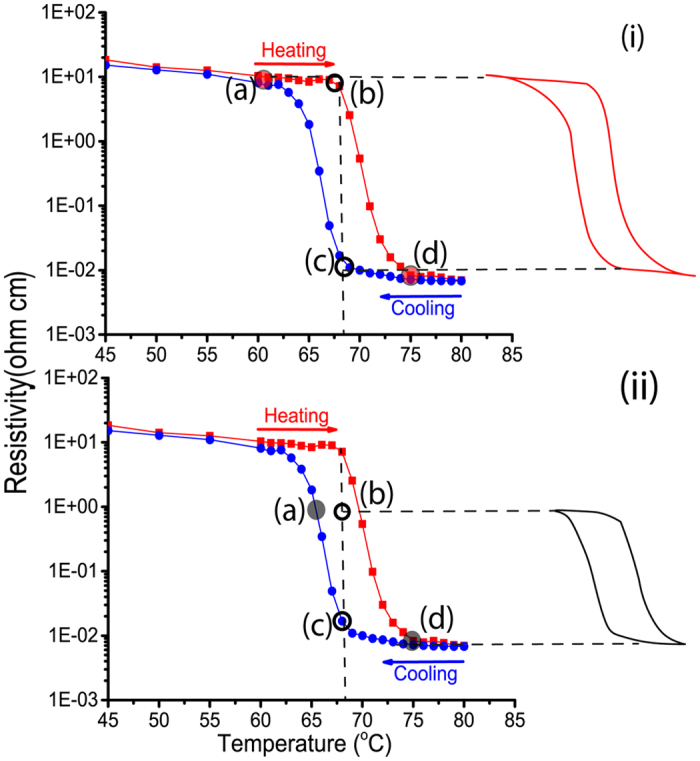
Schematic representation of the thermal switching process based on the hysteresis curve. The impact of adopting two different amplitudes of the thermal activation is illustrated in (i) and (ii). c → a → b: cooling pulse; b → d → c: heating pulse.

Experimental thermal switching behavior of VO_2_ is displayed in Fig. 9. At the background temperature 67 °C the VO_2_ films feature the disordered metamaterial structure. This temperature is applied using a heating stage while monitoring the electrical resistance. The highly resistive semiconducting state is considered as an “off” state. A 3 seconds heating pulse ΔT drives the coalescence of the metallic rutile domains in the metamaterial, a state that is retained after the back stabilization of the temperature at 67 °C. This behavior originates from the difference between the forward and backward transition temperatures due to hysteresis width as illustrated schematically in Fig. 10. In order to switch the metamaterial back to the “off” state, a cooling pulse for a short duration induces the shrinkage and confinement of the metallic domains. Consequently, the metamaterial features a resistive semiconducting behavior even when it stabilized back at 67 °C. Hence, short thermal activation pulses are reliably implemented for abrupt manipulation of the electrical properties of VO_2_ in the metamaterial state. The amplitude of the thermal pulse activation has a direct impact on the response of VO_2_ metamaterial as shown in Figs 9 and 10.

**Figure 11:**
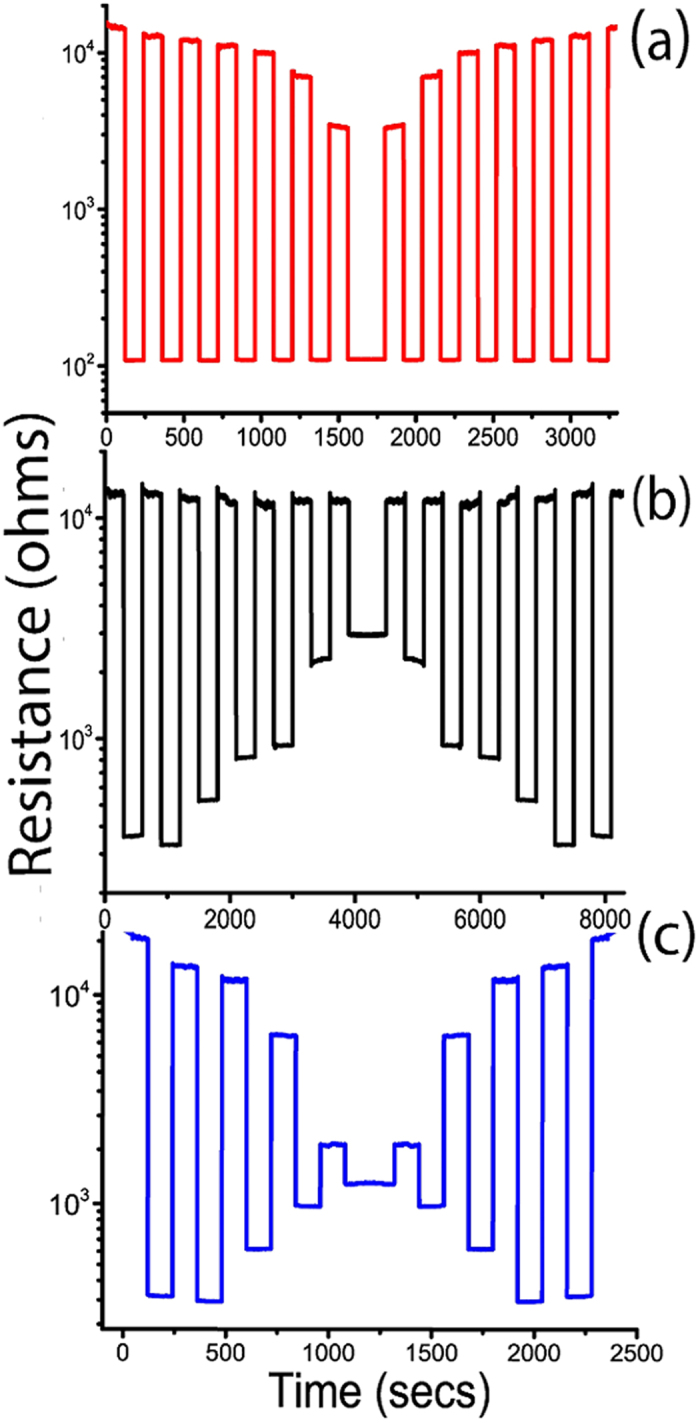
Temperature-programmed resistance switching of the metamaterial via the adjustment of heating (**a**), cooling (**b**) or both heating and cooling (**c**) activation pulses.

Using high strength of the thermal activation pulse as shown in Fig. 10(i) allows the “on” and “off” states of the system at points (b) and (d) respectively, thus taking a high benefit of the resistivity change. Whereas a weaker thermal activation pulse as shown in Fig. 10(ii) restricts the amplitude of switching. This enables programing the metamaterial to follow virtually any particular switching pattern. Few such examples are experimentally performed and the results are displayed in Fig. 11. Adjusting the strength of the heating and/or cooling pulses allows manipulating the resistance-switching pattern. This degree of flexibility proves the robustness and reliability of this thermally triggered VO_2_ electrical switching metamaterial.

It is worth noting that attaining high switching amplitude while implementing a small thermal activation requires a sharp transition with a minimal hysteresis width. The narrow hysteresis, ΔT = 3 K, obtained in this study is equivalent to single crystal or epitaxial films and is ideal for high performance applications^25^,^27^. The self-assembled VO_2_ disordered metamaterial films operate optimally even after extensive and extended use, which makes it an attractive candidate for highly demanding applications. The attractiveness of such metamaterial will substantially enhance, provided a reliable approach is developed for the tuning of the SMT temperature without scarifying its quality in terms of amplitude, sharpness and hysteresis width.

## Conclusions

Indigenous oxidative sintering approach was implemented in this study to induce an efficient densification of the grown VO_2_ film. This multistep process was performed in the same CVD reactor without intermediate handling and air exposure of the samples. The resulting films feature SMT characteristics that match those of epitaxial or bulk VO_2_ in terms of sharpness and width of the hysteresis. This development paves the way towards the exploration and understanding the behavior of this material. Spatially resolved phase analysis using Raman mapping within the transition regime reveals the self-assembly of VO_2_ disordered metamaterial that exhibit an outstanding long-term stability. The formation of locally confined M2 intermediate phase during the transition was attributed to the presence of localized strain. The disordered metamaterial was thermally activated to tune the degree of coalescence/confinement of the metallic domains with high precision. Such a control enables a highly accurate and tunable thermal triggering of the electrical switching.

## Methods

Films of vanadium oxide were deposited on silicon substrates using direct liquid injection MOCVD (MC200 from AnnealSys), which is a stagnation point-flow warm-walled reactor. Cyclohexane solution containing 5 × 10^−3^ mol/l of vanadium (V) oxy-tri-isopropoxide was used as a single-source precursor, which was maintained under nitrogen atmosphere at room temperature before its injection into the evaporation chamber at a frequency of 2 Hz and an adjusted opening time to reach a feeding rate of 1 g/min. The pressure and temperature of the evaporation chamber were maintained at 0.6 mbar and 200 °C during deposition respectively, whereas the walls of the reactor were maintained at 200 °C. During the growth, 500 sccm of nitrogen carrier gas was introduced alongside the precursor injection and the total pressure of the reactor was automatically regulated at 0.6 mbar. The substrate is maintained at 600 °C during the 2 hours of deposition and the subsequent heat treatments.

In a second step, an hour long annealing was performed at 600 °C right after the deposition under oxygen partial pressure of 1 × 10^−2^ mbar. The sample is then further subjected to annealing at the same temperature under vacuum (~0.6 mbar) acting as a reducing atmosphere for 4 hours, after which the chamber is allowed to cool down. All depositions were carried out on 4-inch silicon wafers without removing the upper native oxide layer that might act as a barrier. Samples were later cut into smaller pieces for analysis purposes. Uniform, high quality VO_2_ films were observed throughout the wafers in a homogeneous manner.

### Characterization

X-ray diffraction (XRD), Bruker D8, with CuKα as the X-ray source, was used to identify the crystalline phases of vanadium oxide. Data were collected in the θ−2θ (locked couple) mode from 2θ of 20° to 60° with a step size of 0.02°.

Film thickness was measured using an Alpha step d-500 profilometer from KLA-Tencor and FEI Helios Nanolab 650™, Scanning Electron Microscope (SEM). Surface morphology was inspected by SEM at a working distance of 4 mm with an operating voltage of 25 kV.

Raman spectroscopy was performed using an InVia Raman spectrometer from Renishaw with a 532 nm laser. *In situ* temperature-dependent Raman measurements were performed at ambient air with a Linkam TMS heating stage using fixed heating and cooling ramp at 5 °C/min.

The electrical resistivity was measured using four-point probe measurements in Van der Pauw configuration. Infrared image analysis was conducted using the FLIR X6580SC thermal camera operating in the 1.5–5.1 μm spectral range with an accurate recording at a frequency of 355 Hz in a full 640 × 512 resolution. Temperature-dependent measurements were performed by placing the sample on a heating stage and cycling the temperature form 40 °C to 80 °C while a thermocouple was placed on the sample to measure the surface temperature. The ramp was fixed at 5 °C/min in the transition range.

## Additional Information

**How to cite this article**: Kumar, S. *et al*. Electrical Switching in Semiconductor-Metal Self-Assembled VO_2_ Disordered Metamaterial Coatings. *Sci. Rep.* **6**, 37699; doi: 10.1038/srep37699 (2016).

**Publisher's note:** Springer Nature remains neutral with regard to jurisdictional claims in published maps and institutional affiliations.

## Supplementary Material

Supplementary Information

Suplemtary video
