# Supplementary material for: Electrical Switching in Semiconductor-Metal Self-Assembled VO2 Disordered Metamaterial Coatings
Source: Sci Rep. 2016 Nov 24;6:37699. doi: 10.1038/srep37699 (PMC5121613; doi:10.1038/srep37699)
Supplement: Supplementary Information [file srep37699-s1.doc]

# Supplementary Information

# Electrical switching in semiconductor-metal self-assembled VO_2_ disordered metamaterial coatings.

**Sunil Kumar ^1^, Francis Maury ^2^, Naoufal Bahlawane ^1^.**

^1^Luxembourg Institute of Science and Technology (LIST), 5 avenue des Hauts-Fourneaux L-4362 Esch-sur-Alzette Luxembourg.

^2^CIRIMAT, ENSIACET-4 allée E. Monso, 31030 Toulouse, France

The oxidation of the as-grown VO_2_ to form V_2_O_5_ occurs already after short heating at 400°C. The increase of the diffraction peaks intensity and their narrowing highlight the considerable improvement of the V_2_O_5_ crystallinity with the increase of the temperature.


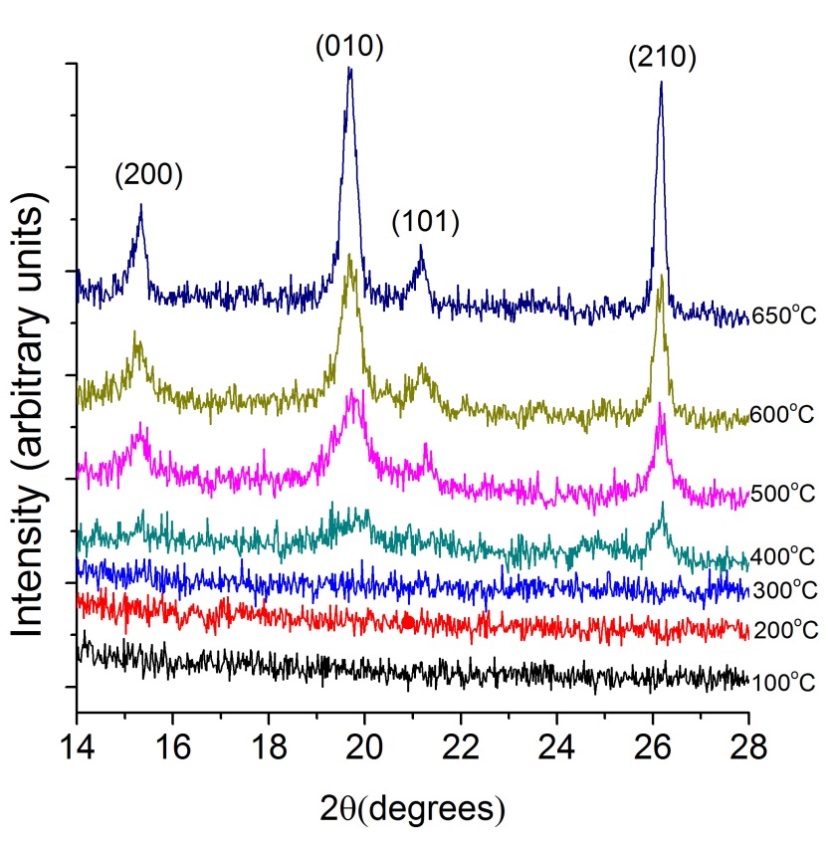


Figure S1. XRD patterns showing the oxidative conversion of the as-grown amorphous VO_2_ into crystalline V_2_O_5_ (PDF no 750457) as a function of temperature.

Structural phase transition is studied by tracking the changes in peak position from X-ray diffraction data as shown in figure S2. It is interesting to notice an apparent peak shift from monoclininc to rutile occurring abruptly over a narrow temperature range. However no trace of M2 phase was detected (mains peaks expected at 27.4° and 28.3° after PDF 01-076-0673), which might be due to the small fraction of M2 when compared to M1. In this condition, it is likely that the implemented XRD technique is not able to capture the weak M2 signal.


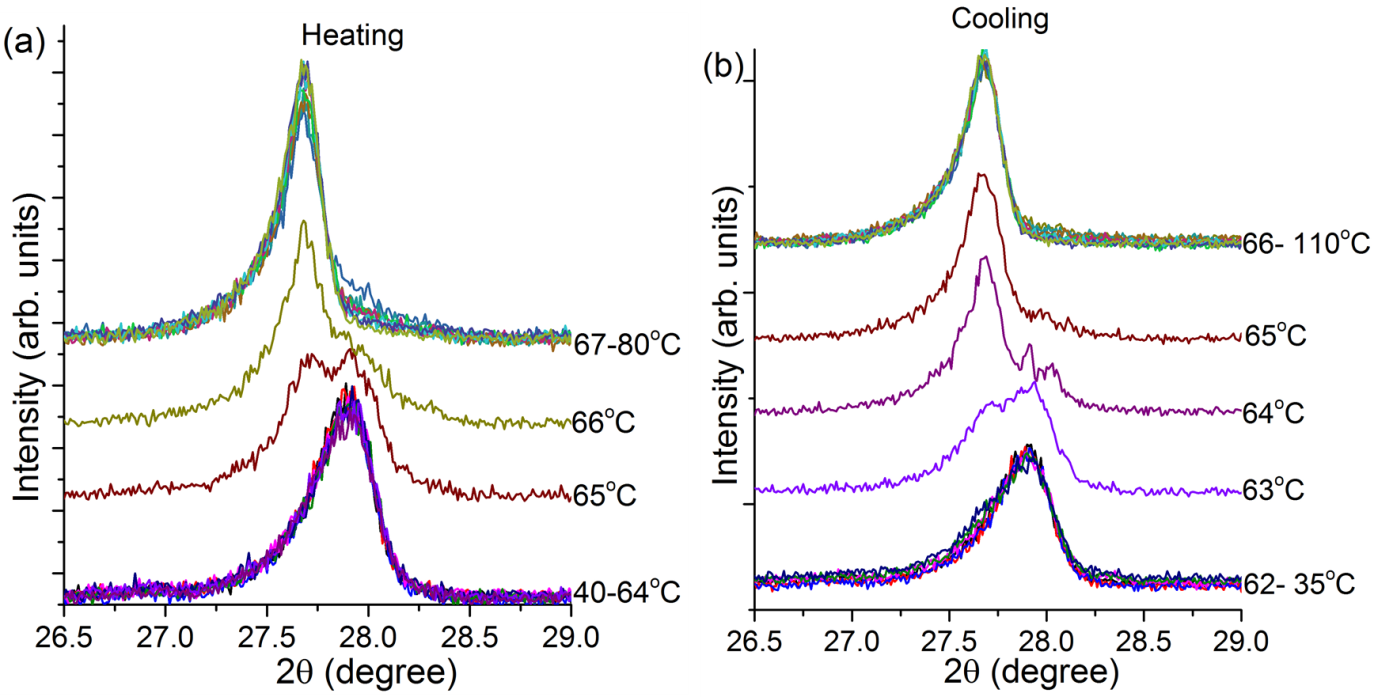


Figure S2. XRD patterns showing the peak shift from monoclinic to rutile and vice versa by (a) heating and (b) cooling the sample. Notice the change in the peak position from 27.9^o^ to 27.6^o^ before and after transition from monoclinic to Rutile phase.

The presence of the M2 phase could be captured using Raman spectroscopy as shown in figure S3. Although the overall Raman signature seems similar between the M1 and M2 phases, a shift of several peaks can be highlighted. The most prominent shift is observed for the peak at 620-650 cm^-1^. On the same surface and at a distance some micrometers, it is possible to identify spots where the SMT occurs directly from the M1 to the R phase and others where the transition is mediated by the M2 phase.


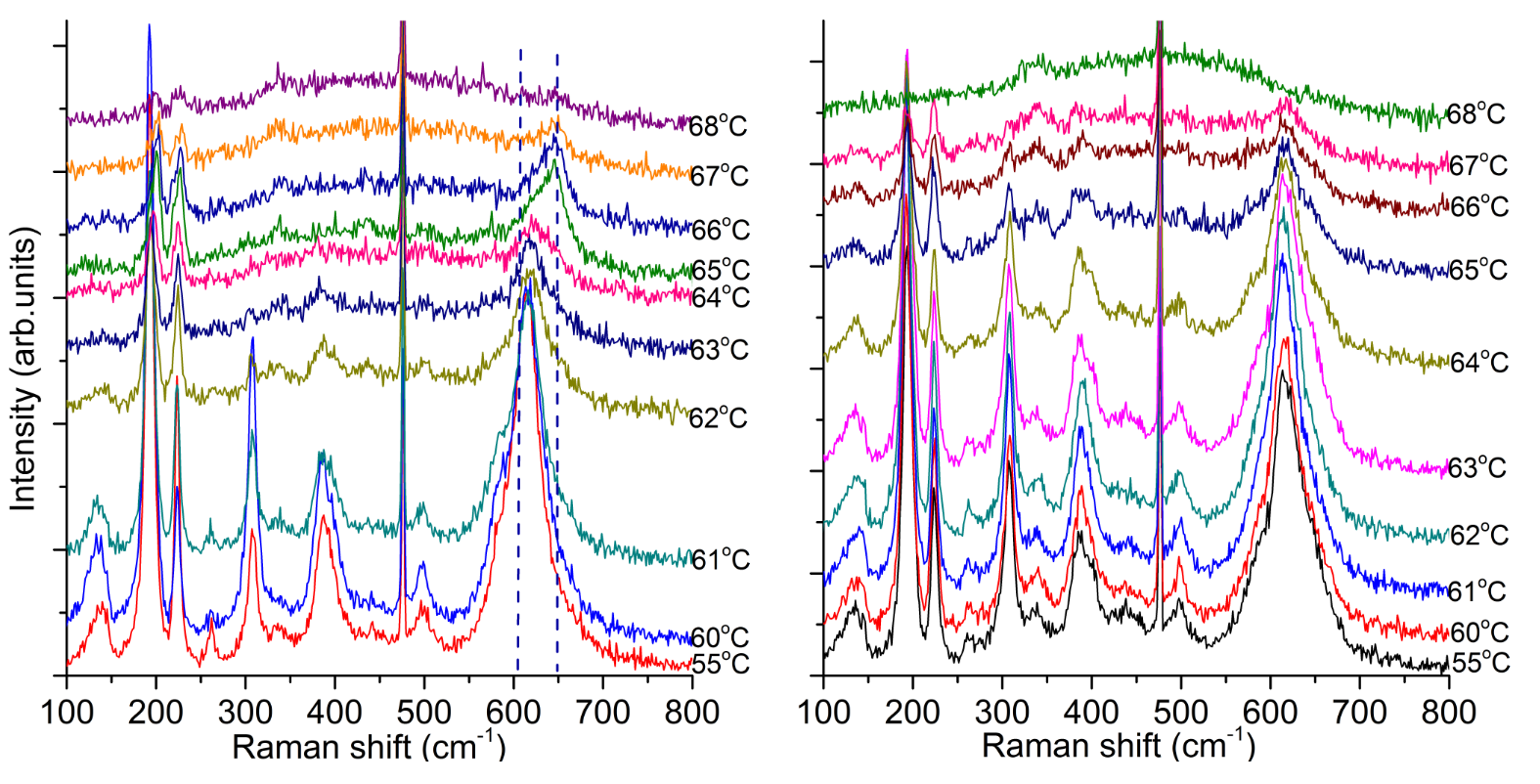


Figure S3. Temperature-programmed Raman analysis during the cooling stage in two close positions on the surface of VO2 film. The left-hand panel represents data from an analysis spot that shows an M1-M2-R transition path, whereas the right-hand panel shows a neighboring position on the same sample that shows an M1-R transition.

**Video legend:**

**Dynamics of the semiconducting-to-metal transition with infrared imaging. The formation of metallic domains and their growth is visualized with increased temperature, while the reverse effect , confinement and vanishing, occurs during the cooling stage.**
